# A prediction model to identify hospitalised, older adults with reduced physical performance

**DOI:** 10.1186/s12877-017-0671-5

**Published:** 2017-12-07

**Authors:** Inge H. Bruun, Thomas Maribo, Birgitte Nørgaard, Berit Schiøttz-Christensen, Christian B. Mogensen

**Affiliations:** 1 Department of Physiotherapy, Lillebaelt Hospital, University of Southern Denmark, Kolding, Odense, Denmark; 2 Department of Regional Health Research, University of Southern Denmark, Odense, Denmark; 3 Department of Public Health, Aarhus University, Aarhus, Denmark; 4 DEFACTUM, Central Denmark Region, Aarhus, Denmark; 5 Department of Public Health, University of Southern Denmark, Odense, Denmark; 6 Spine Centre of Southern Denmark, Lillebaelt Hospital, University of Southern Denmark, Odense, Denmark; 7 Emergency Department, Hospital of Southern Jutland, University of Southern Denmark, Odense, Denmark

**Keywords:** Reduced physical performance, Prediction model, Physical activity

## Abstract

**Background:**

Identifying older adults with reduced physical performance at the time of hospital admission can significantly affect patient management and trajectory. For example, such patients could receive targeted hospital interventions such as routine mobilisation. Furthermore, at the time of discharge, health systems could offer these patients additional therapy to maintain or improve health and prevent institutionalisation or readmission. The principle aim of this study was to identify predictors for persisting, reduced physical performance in older adults following acute hospitalisation.

**Methods:**

This was a prospective cohort study that enrolled 117 medical patients, ages 65 or older, who were admitted to a short-stay unit in a Danish emergency department. Patients were included in the study if at the time of admission they performed ≤8 repetitions in the 30-s Chair-Stand Test (30s–CST). The primary outcome measure was the number of 30s–CST repetitions (≤ 8 or >8) performed at the time of follow-up, 34 days after admission. Potential predictors within the first 48 h of admission included: age, gender, ability to climb stairs and walk 400 m, difficulties with activities of daily living before admission, falls, physical activity level, self-rated health, use of a walking aid before admission, number of prescribed medications, 30s–CST, and the De Morton Mobility Index.

**Results:**

A total of 78 (67%) patients improved in physical performance in the interval between admission and follow-up assessment, but 76 patients (65%) had persistent reduced physical performance when compared to their baseline (30s–CST ≤ 8). The number of potential predictors was reduced in order to create a simplified prediction model based on 4 variables, namely the use of a walking aid before hospitalisation (score = 1.5), a 30s–CST ≤ 5 (1.8), age > 85 (0.1), and female gender (0.6). A score > 1.8 identified 78% of the older adults who continued to have reduced physical performance following acute hospitalisation.

**Conclusion:**

At the time of admission, the variables of age, gender, walking aid use, and a 30s–CST score ≤ 5 enabled clinicians to identify 78% of older adults who had persisting reduced physical performance following acute hospitalisation.

**Trial registration:**

ClinicalTrials.gov Identifier: NCT02474277. (12.10.2014).

**Electronic supplementary material:**

The online version of this article (10.1186/s12877-017-0671-5) contains supplementary material, which is available to authorized users.

## Background

Activities of daily living are essential for maintaining independence and for participating in meaningful activity. For older adults, and especially frail, older adults, hospitalisation poses a risk of triggering persistent functional decline, largely by ushering in a period of reduced activity [1–3]. Despite this foreknowledge, older adults who are admitted to medical departments continue to spend more time lying in bed than sitting, standing, or walking [4–7].

The ageing process entails a loss of muscle mass, followed by reduced physical performance and functional decline [1, 8]. In order to mitigate the risk of accelerating this process, it is important to identify frail, older adults at or near the time of hospital admission. This would permit the application of targeted hospital interventions, such as routine patient mobilisation, that can be used to prevent physical decline. Furthermore, at the time of discharge, health systems can elect to offer such patients additional therapy or supports with the intent of maintaining and improving health and preventing institutionalisation or readmission.

However, identifying such patients is challenging, largely because valid information on previous physical performance level is often lacking.

Existing screening tools used at the time of admission focus primarily on adverse outcomes such as readmission and functional decline [9]. They have shown limited reliability [9] and are based entirely on self-reported information [10]. At a hospital level, self-reported information provides important information on previous functioning, but older adults often overestimate their own functional abilities [11, 12].

The 30-s Chair-Stand Test (30s–CST) and a cut-off point of 8 repetitions can predict the loss of functional mobility in older, community-dwelling adults [13]. Furthermore, physical performance measures have demonstrated predictive ability in acute, admitted older adults [14–21]. While a prediction model based solely on physical performance can lead to misclassification, since performance often improves from admission to discharge [17, 18], it remains true that most older adults with reduced physical performance at the time of admission continue to have poor performance at discharge [22].

This study aimed to identify predictors for persisting, reduced physical performance in older adults following acute hospitalisation.

The objectives were: 1) to describe changes in physical performance in older adults from admission until a minimum of 14 days after admission; 2) to identify potential predictors at admission for those older adults who have persistent reduced physical performance following hospitalisation; and 3) to develop a simple prediction model that will enable clinicians to identify at the time of admission those older adults who will continue to have reduced physical performance following acute hospitalisation.

## Methods

### Study design and participants

A prospective cohort study was conducted in a short-stay unit in a Danish emergency department (ED) from December 2014 to May 2015 [23]. In Denmark a short-stay unit provides targeted care for 48–72 h, followed by patient discharge or transfer to an in-patient unit. All participants were enrolled consecutively and assessed within the first 48 h of admission and again at a follow-up home visit that took place a minimum 14 days after the date of admission.

We recruited patients ages 65 years or older who were admitted to the short-stay unit, who resided in the hospital’s catchment area, and who were admitted with a medical diagnosis (rather than a surgical or psychiatric diagnosis). Common medical diagnoses included infection, thromboembolic disease, musculoskeletal disease, and cardiovascular disease, but not patients with obvious signs of stroke or ST-elevation myocardial infarctions. Patients were enrolled in the study if they demonstrated reduced physical performance within the first 48 h of admission, specifically if they performed ≤8 repetitions in 30s–CST. We assumed that older adults who performed >8 repetitions in the 30s–CST were without significant risk of losing functional mobility, and hence the rationale for their exclusion from the study. Additional inclusion criteria included patient ability to sit on a chair independently within the first 48 h of admission, patient orientation to time and place, and patient ability to speak and understand Danish. Patients who could not walk at their baseline health were excluded.

### Outcome measurement

The sole study outcome measurement was the 30s–CST. Older adults with a 30s–CST ≤ 8 were classified as having reduced physical performance, whereas those with a 30s–CST > 8 were considered to have non-reduced physical performance. The cut-off point was chosen based on evidence that community-dwelling older adults scoring ≤8 in the 30s–CST are at risk of losing functional mobility. This cut-off point was deemed to have acceptable validity and reliability [13, 24].

### Potential predictors

The following self-reported information was collected in the process of evaluating potential predictors of persistent, reduced physical performance: age, gender, and mobility (climbing stairs and walking 400 m) [25]. Patients were asked if they had experienced difficulties with activities of daily living (ADL) within the last 2 weeks before the admission [26], if they had experienced falls, if they had participated in moderate physical activity (excluding ADLs) that was strenuous enough to increase work of breathing and pulse, how they perceived their health [27], and finally if they had used a walking aid before admission [28]. Additional potential predictors included the number of prescribed medications (taken from medical records) and physical performance as assessed by the 30s–CST and the De Morton Mobility Index (DEMMI) [29].

The 30s–CST assesses lower-body strength and has moderate inter-rater reliability for acute, admitted ‘medical’ patients. A floor effect at the time of admission makes the test only moderately feasible in an acute care setting, but on the other hand the simplicity of the test facilitates its use in a busy, short stay unit [15]. The 30s–CST was performed by counting the number of times in a 30 s interval that a patient can stand from a sitting position with their hands crossed against their chest [30]. A Minimum Importance Change (MIC) on 2.9–2.6 stands has been determined for the 30s–CST [31].

DEMMI assesses mobility and balance through 15 hierarchical items and provides a score between 0 and 100 [29]. DEMMI is a valid and reliable measurement of these parameters for both hospitalised and community-dwelling older adults [14, 32–34]. A Minimal Detectable Change MDC_90_ of 9.0 and a Minimal Clinically Important Difference (MCID) of 10.0 has been determined for DEMMI [32].

Information on living arrangement, education, acute diagnosis, destination after ED (home or another department), and contact with social services before hospitalisation was collected either as self-reported information or from medical records, and used as demographic factors. Cognitive performance was tested using the Orientation–Memory–Concentration Test (OMC) [35].

### Procedure

On weekday mornings, a physiotherapist recruited and tested patients for eligibility. Included patients provided written consent for study enrolment. In the 30s–CST assessment, patients who were unable to stand with their hands crossed against their chest scored 0. Patients who completed the task in a practice test, but were unable to stand in the actual test scored 1. To avoid fatigue after the 30s–CST test, we collected self-reported information before testing patients with the DEMMI. The DEMMI protocol was followed, except for the ‘sit to stand no arms’ (DEMMI item 6), as this had been demonstrated in the 30s–CST. After data collection, there was no further contact between the patient and the physiotherapist. The health staff had no access to study data and treatment was unaffected by study participation.

To inoculate post-discharge physical performance assessments from bias, a second physiotherapist, who did not perform the initial assessment, was selected to perform the follow-up assessment. If the patient was unable to participate at the originally scheduled post-hospital assessment then a later visit was scheduled soon thereafter. At the follow-up assessment, the 30s–CST and DEMMI were conducted with a ten-minute break between tests.

### Statistical methodology

The sample size was calculated on the following assumption: for a multivariate analysis of potential predictors *n* = 50 + 8×, where x is the number of independent variables [36]. Since we anticipated a 20% dropout rate, a total of 156 patients were required as a precondition to including 10 potential predictors.

Potential predictors were classified into the following five domains: 1) demographic: age and gender; 2) self-reported mobility: walking 400 m, climbing a flight of stairs, walking aid use before admission, and falls; 3) self-reported habitual physical status: physical activity, self-rated health, and difficulties with ADLS in the 2 weeks before admission; 4) polypharmacy: number of prescribed medications; and 5) presenting physical performance: the 30s–CST and the DEMMI at the time of admission.

For the univariate logistic regression analysis, all continuous variables were dichotomised, with the exception of age, which was classified into 5 levels given the known association between age and physical performance. Cut-off points for continuous variables were based on Receiver Operating Characteristics (ROC) for the study data and on a literature review. In the literature we found a relationship between the ability to rise a maximum of five times and the risk of sarcopenia [37] and polypharmacy, as defined by ≥10 drugs associated with physical performance [38]. We found no recommended cut-off points for DEMMI. However, for semi-independent community-dwelling seniors, a score of 76.5 (95% CI 73.1–79.9) had previously been reported [39]. The ROC analysis revealed cut-off points at 30s–CST = 5, polypharmacy = 16 and DEMMI = 57 (see Additional file 1). We used the cut-off points found in the literature, except for DEMMI, for which the ROC cut-off 57 was used on account of the fact that acutely hospitalised older patients have lower physical performance than community-dwelling older adults [40]. Factors on ordinal scales were dichotomised (without difficulty or with difficulty/not at all).

For the multivariate analysis, age and gender were preselected [13, 17, 39–42]. The smallest numbers of events determined the permitted number of predictors [43]. Potential predictors were included in the multivariate analysis using the following data reduction: 1) potential predictors with a *p* value ≤0.20 in the univariate analysis were considered [44]; 2) if the predictors within a domain had a moderate (>0.50) correlation, the potential predictor with the highest odds ratio was selected; 3) the final selection of predictors was based on the odds ratios and the assumed ease of use in an ED setting.

The potential predictors were tested for interaction. The area under the curve (AUC) was used to identify the final model and the model was tested with Hosmer–Lemeshow and for internal validity by bootstrapping [45].

Beta coefficients were employed to calculate the total score. The prediction model’s performance was assessed by calculating the sensitivity/specificity and predictive values for older adults with continuous reduced physical performance upon follow-up. Moreover, we identified the number needed to treat/test (NNT).

Analyses were performed using STATA 14 (Stata Statistical Software, College Station, TX) in adherence with principles outlined in the guidelines for Strengthening the Reporting of Observational Studies in Epidemiology [46] and Transparent Reporting of a Multivariable Prediction Model for Individual Prognosis or Diagnosis [47].

The Regional Scientific Ethical Committees of Southern Denmark approved this study with a waiver (20.08.2014). As required by Danish legislation, written informed consent was obtained from participants to permit collection of information from medical records. The project was registered with the Danish Data Protection Agency (2008–58-0035) and in the ClinicalTrials.gov Identifier: NCT02474277 (12.10.2014).

## Results

**Fig. 1:**
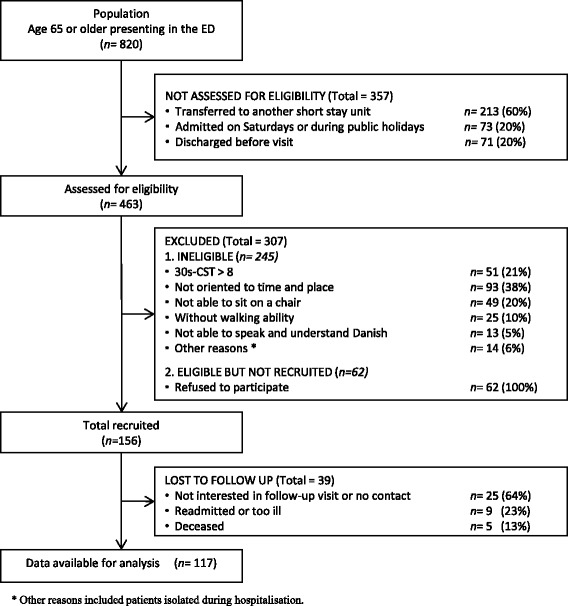
Flow chart of inclusion process

Overall, 820 older adults were admitted to the ED during the recruitment period and 156 patients were included in the study. A flowchart of inclusion, reasons for exclusion, and loss to follow-up appears in Fig. 1.

The follow-up occurred median 34 days (IQR 27–40 days) after admission. A total of 39 (25%) of the enrolled patients dropped out of the study prior to their follow-up assessment, leaving 117 patients for further analysis.

An analysis of patients who were lost to follow up compared to those who completed the study did not reveal significant differences in the examined variables, with the exception that 25 of the 39 (64%) patients who were lost to follow up did not walk independently at baseline compared with 50 of the 117 (43%) patients who completed the study (*p* = 0.02)*.*

The basic characteristics of the enrolled patients are provided in Table 1, as are their admission characteristics in accordance with a 30s–CST ≤ 8 or >8 at the follow-up visit.

**Table 1:** Cohort characteristics at the time of admission

				Admission characteristics by outcome status at follow-up			
		All participants (*n* = 117)		30s–CST >8 (*n* = 41)		30s–CST ≤8 (*n* = 76)	
Self-reported information		n	%	N	%	n	%
Living arrangement	Alone	66	56	22	54	44	58
	Cohabition	50	43	19	46	31	41
	Nursing home	1	1			1	1
Education	No vocational education	49	42	17	41	32	42
	Vocational or short-term training	53	45	13	32	40	53
	Medium/long/other education	15	13	11	27	4	5
Physical performance measures		median	IQR	median	IQR	median	IQR
30s–CST^a^		0	(0–5)	5	(0–7)	0	(0–2)
DEMMI^b^		44	(33–62)	62	(39–67)	41	(27–53)
Cognitive level		median	IQR	median	IQR	median	IQR
OMC^c^ (*n* = 104)		24	(20–26)	24	(22–28)	23	(18–26)
Basic Mobility		n	%	N	%	n	%
Unable to rise with hands crossed against the chest		48	41	28	68	20	26
Unable to walk independently		50	42	12	29	38	50
Able to walk with walking aid		32	27	8	20	24	32
Able to walk without walking aid		35	30	21	21	14	18
Extracted information		n	%	N	%	n	%
Discharged from ED to home		51	44	19	46	32	42
Discharged from another department		66	56	22	54	44	58
Presenting complaints^d^	respiratory disorder	20	23	7	23	13	23
All participants (*n* = 87)	fever	16	18	7	23	9	16
30s–CST > 8 (*n* = 31)	nonspecific illness	13	15	4	13	9	16
30s–CST ≤ 8 (*n* = 56)	emergency track	12	14	5	16	7	13
	diarrhoea and/or vomiting due to infection	5	6	2	6	3	5
	extremity pain	3	4			3	5
	pain or disease in urinary tract	3	4	2	6	1	2
	dizziness	3	4			3	5
	chest pain	2	2			2	4
	head pain	2	2	2	6		
	others, including falls	8	9	2	6	6	11

^a^ 30-s Chair-Stand Test, ^b^ De Morton Mobility Index (0–100), ^c^ Orientation-Memory-Concentration Test (0–28)

^d^ Presenting complaints were extracted from a central database, these depend on doctor’s report

Overall, the median age was 77 years (IQR 71–85 years) and 68 (58%) were females.

Patients who demonstrated reduced physical performance at the time of follow-up were older (78 years; IQR 72–86) than those patients who had non-reduced physical performance (75 years; IQR 70–80). Approximately one third of patients enrolled in the study did not receive home health care from the municipality.

As a group, the mean length of stay (LOS) was 4.3 (SD 3.8) days. Patients discharged from the short stay unit had a mean LOS of 1.9 (SD 1.8), whereas patients transferred to a different ward had a mean LOS of 6.2 (SD 4.0) days. Further comparison between patients discharged from the short stay unit and patients transferred to a different ward showed that the former cohort had better performance testing at admission than the latter. For patients discharged or transferred to other wards the median 30s–CST scores were 2 (IQR 0–6) and 0 (IQR 0–3), respectively. At follow-up 63% of the patients discharged from the short stay unit had a 30s–CST ≤ 8 and 67% of patients transferred to other wards had a 30s–CST ≤ 8.

### Changes in physical performance

Altogether, 78 (67%) of the patients improved their 30s–CSTs from admission to follow-up, 35 (30%) had an unchanged 30s–CST, and 4 (3%) had a lower 30s–CST. Although most patients improved from admission to follow-up, 76 (65%) of patients demonstrated persisting reduced physical performance (30s–CST ≤ 8).

More than half of patients had a 30s–CST improvement of 5 (IQR 3–7.3). The improvement was substantial for a sub-set of 13 patients (19%): their 30s–CST was 0 at admission and 11 at follow-up (IQR 10–12).

For DEMMI, 88 (75%) of the patients demonstrated improvements, whereas 16 (14%) deteriorated. The median improvement was 18.5 points (IQR 10.3–32.5).

### Potential prognostic factors associated with reduced physical performance

Univariate analysis revealed 10 potential predictors with a *p* value ≤0.20; these were selected for further analysis (Table 2). The correlation was >0.50 or in other words of moderate strength, within the study domains of self-reported physical performance and presenting physical performance (see Additional file 2). This left six potential predictor variables for further model development, namely climbing stairs, physical activity, self-rated health, walking aid use, polypharmacy, and the 30s–CST, in addition to the preselected variables of age and gender.

**Table 2:** Potential predictors for reduced physical performance (30s–CST ≤ 8) at follow-up (*n* = 117)

					Univariate analysis			Multivariate analysis			Bootstrapping	
Potential predictors	30s–CST > 8 (*n* = 41)	%	30s–CST ≤ 8 (*n* = 76)	%	Odds Ratio	95% CI	*p*-value	Odds Ratio	95% CI	*p*-value	95% CI	*p*-value
*Domain: Demographic*												
Age (years)												
65–70	10	24	13	17	1							
71–75	10	24	18	24	1.4	0.4–4.3	0.57					
76–80	8	20	11	14	1.1	0.3–3.6	0.93					
81–85	8	20	13	17	1.3	0.4–4.2	0.72					
> 85	5	12	21	28	3.2	0.9–11.7	0.07	1.1	0.3–4.2	0.88	0.3–4.8	0.89
Gender												
Male	21	51	28	37	1							
Female	20	49	48	63	1.8	0.8–3.9	0.14	1.8	0.7–4.5	0.20	0.7–4.9	0.25
*Domain: Mobility*												
Climbing a flight of stairs												
Without difficulty	29	71	25	33	1							
With difficulty/not at all	12	29	51	67	4.9	2.2–11.3	<.001					
Walking 400 m												
Without difficulty	27	66	31	41	1							
With difficulty/not at all	14	34	45	59	2.8	1.3–6.2	0.01					
Use of walking aid (in/outdoors)												
Not at all	31	76	23	30	1							
Sometimes/all the time	10	24	53	70	7.1	3.0–17.0	<.001	4.4	1.6–12.0	0.003	1.4–14.2	0.01
Falls												
No falls	28	68	57	75	1							
One or more falls	13	32	19	25	0.7	0.3–1.7	0.44					
*Domain: Habitual physical status*												
Participation in physical activity												
More than once a week	20	49	14	18	1							
Not at all	21	51	62	82	4.2	1.8–9.8	<.001					
Self-rated health (*n* = 116)												
Excellent/very good/good	33	82	43	57	1							
Less good/poor	7	18	33	43	3.6	1.4–9.2	0.01					
Difficulties in ADL												
not at all	18	44	29	38	1							
Some/most of the time	23	56	47	62	1.3	0.6–2.8	0.55					
*Domain: Polypharmacy*												
Polypharmacy												
< 10	28	68	39	51	1							
≥ 10	13	32	37	49	2.0	0.9–4.5	0.08					
*Domain: Presenting physical performance*												
30s–CST^a^												
Score > 5	18	44	6	8	1							
Score ≤ 5	23	56	70	92	9.1	3.2–25.9	<.001	5.8	1.9–17.8	0.002	1.5–21.9	0.01
DEMMI^b^												
Score > 57	21	51	12	16	1							
Score ≤ 57	20	49	64	84	5.6	2.3–13.4	<.001					

^a^ 30-s Chair-Stand Test,^b^ De Morton Mobility Index (0–100)

Hosmer-Lemeshow 0.19

The final selection of predictors, based on the odds ratio and the anticipated applicability and feasibility of use in the ED setting, narrowed down potential predictors to walking aid use before hospitalisation (OR: 7.1) and the 30s–CST ≤ 5 (OR: 9.1).

No significant interactions were found between potential predictors and the outcome measurement. The AUC for the full model was 0.80 (95% CI: 0.72; 0.89). The multivariate analyses showed that walking aid use before hospitalisation had an OR of 4.4 and that a 30s–CST ≤ 5 had an OR of 5.8 (Table 2).

### A simple prediction model

Table 3 presents the selected predictors and their beta coefficients. In this sample, a score > 1.8 upon admission was able to identify 78% of patients who continued to have a reduced physical performance 1 month after acute hospitalisation. Furthermore, using a score of >1.8 only 2.43 patients were needed to identify one patient with reduced physical performance at follow-up (number needed to test).

**Table 3:** Prediction model to identify patients with persistent reduced physical performance after hospitalisation

Predictors	Beta coefficient			
Age > 85 years			0.1	
Female gender			0.6	
Use of walking aid (in−/outdoors)			1.5	
30s–CST ≤ 5			1.8	
Total score			4.0	
	Sensitivity (95% CI)	Specificity (95% CI)	Positive predictive value (95% CI)	Negative predictive value (95% CI)
Prediction model (cut-off >1.8)	82% (71–90)	59% (42–74)	78% (68–87)	63% (46–78)

## Discussion

In this study, the majority of acutely admitted older adults identified with a 30s–CST score ≤ 8 at admission improved their physical status by the time of study follow-up. However, almost two thirds continued to have reduced physical performance (30s–CST ≤ 8). Several self-reported information and physical performance variables were associated with persistently reduced physical performance. On admission, a prediction model based on age, gender, walking aid use (indoor or outdoor) before hospitalisation, and a 30s–CST ≤ 5 allowed the authors to identify 78% of the older adults who continued to have reduced physical performance 1 month after admission.

### Changes in physical performance

Our finding, that a majority of patients improved their physical performance from the time of admission to 1 month after admission, corroborates the findings from earlier studies that used the Short Physical Performance Battery (SPPB) and walking speed [17, 18, 22]. In our study 65% of patients showed reduced physical performance 1 month after admission, reinforcing the need to provide this group with targeted interventions, since frailty is associated with a loss of independence, increased community costs, and readmission [13, 18, 48].

### Potential prognostic factors associated with reduced physical performance

The univariate logistic regression revealed ten potential predictors for reduced physical performance (*p* value ≤0.20). Besides the preselected variables of age and gender, the event rate allowed two potential predictors to be included in the multivariate analysis. We selected use of walking aid before hospitalisation and a 30s–CST ≤ 5, as they had the highest odds ratio and were judged the most feasible tools to use in a busy ED setting. Moreover, using walking aids as a predictor makes clinical sense, since community-dwelling older adults use walking aids to improve balance and mobility [49]. On the other hand, walking aids are risk factors for low mobility [50] and their use before hospitalisation thus implies physical limitations and a higher risk of losing physical ability. Walking aids were also included in Hoogerduijn et al.’s model for assessing the risk of functional decline in acutely hospitalised older adults [51]. The other predictors in that study were a preadmission need for assistance in instrumental activities of daily living, a need for assistance in travelling, and a lack of education after age 14 [51].

### A simple prediction model

We found that gender, age, self-reported information on walking aid use, and a 30s–CST ≤ 5, correctly identified patients who had continued reduced physical performance following acute hospitalisation. Moreover, a score > 1.8 identified 78% of patients with continuous reduced physical performance with a NTT of 2.43 patients. Clinically, all predictors need to be considered, since in isolation none of the model’s variables have a score > 1.8. Our prediction model based on physical measures and self-reported information is the first of its kind. However, a study in primary care settings concerning community-dwelling older adults aged 65 or older has shown that for older adults with poor health the combination of physical performance measures and self-reported information is substantially better than either alone [52].

Existing screening tools to identify older adults who need a comprehensive geriatric assessment (CGA) have shown poor reliability in an acute setting [9]. Our prediction model supports the identification of older adults who could benefit from a CGA, where a functional assessment is an integral part [53]. For patients discharged to other units than the geriatrics unit, the identification of older adults with persistent reduced physical performance might give rise to a targeted hospital intervention such as routine patient mobilisation. Furthermore, this study supports the evidence from other studies that self-reported information and physical performance measures provide different and complementary information [8]. From admission to follow-up, 19% of the patients had a 30s–CST change from 0 to 11. Hence, if the prediction model had been solely based on physical performance then 19% of patients would have been misclassified. For every 2–3 patients tested, clinicians will identify one patient with reduced physical performance 1 month after hospitalisation. However, since the negative predictive value is only 63%, every third with a negative test will still be at risk (Table 3). The prediction model does not comprehensively identify all at-risk patients, which the clinicians should be aware of. Clinically, this prediction model is easily applied: age and gender are known, determining walking aid use before hospitalisation requires one simple question, and the 30s–CST is easy to execute.

### Strength and limitations

The study strength lies in its ability to assess physical performance using a simple objective measurement in combination with self-reported information. We used the 30s–CST in the prediction model while recognising that the floor effect could affect the baseline assessment. This choice was related to the well-known improvement in physical performance measures from admission to discharge [17, 18, 22].

Although up to 48 h was permitted from the time of admission to the time of baseline assessment, in practice the timeframe was much shorter as assessments were performed routinely every weekday morning. It follows that the prediction model was less influenced by the cause of hospitalisation.

The cohort included patients discharged from the short stay unit as well as patients transferred to other wards; thus a different risk for deterioration due to varied length of stay. However, the number of patients with reduced physical performance at follow-up in both groups (discharged from short stay unit or transferred to other wards) was comparable. This lack of difference in deterioration can be explained by the tiredness older adults generally experience after an acute admission [53].

The 30s–CST was used as an outcome measure even though the cut-off point of ≤8 for the 30s–CST is only validated for use in active, community-dwelling, older adults. We did so since the follow-up visit was performed in the older adult’s home.

The binary stratification of the outcome measure might have resulted in a misclassification of some patients, due to the variation in patient performance [54]. We chose this dichotomisation since it is used in current literature [13, 24] and since it reflects recommendations made in Denmark and elsewhere for screening programs for community-dwelling, older adults.

We have described the predictor selection in detail, making the selection process easily reproducible in other settings. We managed to reach our pre-calculated sample size, but the study is weakened by a lower event rate than expected, which in turn restricted the number of predictors that were included in the model. Thus, before clinical implementation we recommend that the model’s external validity is verified through larger studies using a different population. Moreover, the prediction model can only be generalised to older ‘medical’ patients who are mentally fit and show reduced physical performance upon admission.

Patients who were not assessed for eligibility can be seen as introducing a selection bias. However, 55% of patients were excluded based on organisational limitations, such as the day of admission, since patients were only recruited on weekdays. Of note, patients admitted on Sundays were included if they fell within the 48-h limit for enrolment. Patients who refused to participate generally offered two reasons; either they felt the project was irrelevant to them or they did not have the energy to participate.

The follow-up visits were completed at a median of 34 days (IQR 27–40) after admission, although the initial intention was to perform follow-up 14 days after admission. Delays in the follow-up assessment were due to patient preference, patient schedules, and the fact that some patients had not been discharged at the time of planned follow-up. We assume that the delay in follow-up was beneficial for this particular study, since it can be assumed that physical performance would have stabilised over a longer interval of time.

## Conclusion

To minimize the risk for functional decline due to inactivity, it is important to identify older ‘medical’ patients with reduced physical performance at the time of admission. This might give rise to targeted hospital interventions, such as routine patient mobilisation, that can be used to prevent physical decline.

The presented model is easy to use in a busy ED, and for every three patients tested, one older adult with continued reduced physical performance following hospitalisation is identified. The model takes into account information on age, gender, and walking aid use before hospitalisation, combined with 30s–CST results.

## Additional files

Additional file 1: ROC analysis. Receiver Operation Characteristic (ROC) for cut-off points. (PDF 103 kb)

Additional file 2: Correlations within the domains. The correlation for climbing stairs, walking 400 m., use of walking aid, physically activity, self-rated health, and the 30s–CST. (PDF 103 kb)

## Abbreviations

30s–CST: The 30-s Chair-Stand Test
ADL: Activities of daily living
AUC: The area under the curve
DEMMI: De Morton Mobility Index
ED: Emergency department
NNT: Number needed to treat
OMC: Orientation–Memory–Concentration Test
ROC: Receiver Operation Characteristics
SPPB: Short Physical Performance Battery
