# Supplementary figures and images for: A prediction model to identify hospitalised, older adults with reduced physical performance
Source: BMC Geriatr. 2017 Dec 7;17:281. doi: 10.1186/s12877-017-0671-5 (PMC5719737; doi:10.1186/s12877-017-0671-5)

## Additional file 1

Receiver Operation Characteristic (ROC) for cut-off points

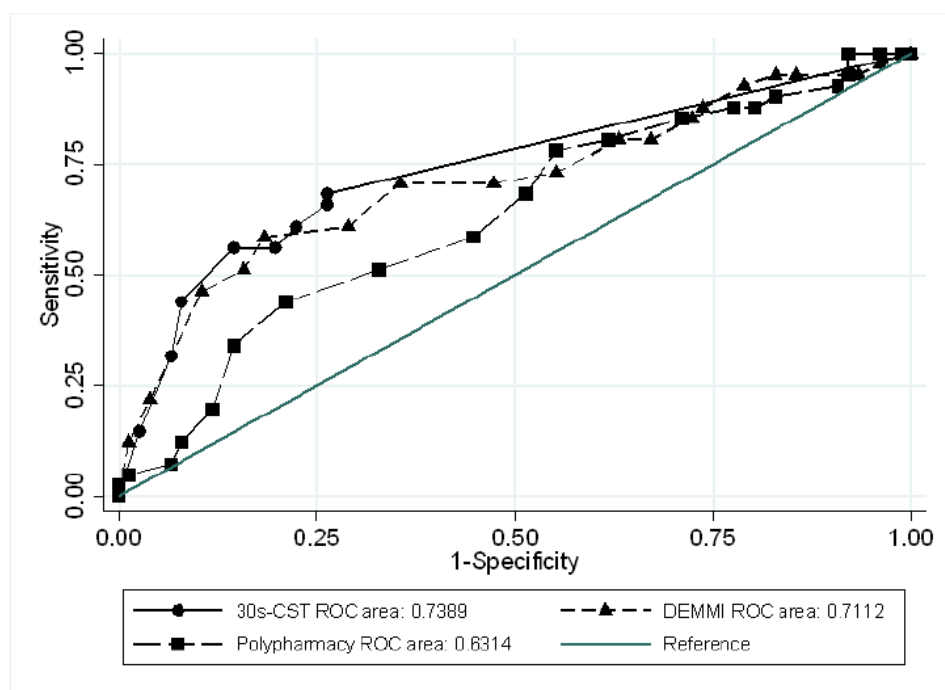

Supplement: Supplementary file 1 — ROC analysis. Receiver Operation Characteristic (ROC) for cut-off points. (PDF 103 kb) [file 12877_2017_671_MOESM1_ESM.pdf]
