# Supplementary material for: A prediction model to identify hospitalised, older adults with reduced physical performance
Source: BMC Geriatr. 2017 Dec 7;17:281. doi: 10.1186/s12877-017-0671-5 (PMC5719737; doi:10.1186/s12877-017-0671-5)
Supplement: Supplementary file 2 — Correlations within the domains. The correlation for climbing stairs, walking 400 m., use of walking aid, physically activity, self-rated health, and the 30s–CST. (PDF 103 kb) [file 12877_2017_671_MOESM2_ESM.pdf]

Additional file 2

	Climbing stairs*	Walking aid *	Physically active ǀ	30s-CST §[2]
Walking 400 m*	r= 0.6	r=0.1		
Walking aid*	r=0.3			
Self-rated health ǀ			r= 0.1	
DEMMI §[1]				r= 0.6

* Within the domain self-reported mobility

ǀ Within the domain self-reported habitual physical status

§ Within the domain presenting physical performance [1] De Morton Mobility Index (0-100)

[2] 30-second Chair- Stand Test
